# Functional analysis of the *TM6* MADS-box gene in the octoploid strawberry by CRISPR/Cas9-directed mutagenesis

**DOI:** 10.1093/jxb/ery400

**Published:** 2018-11-13

**Authors:** Carmen Martín-Pizarro, Juan Carlos Triviño, David Posé

**Affiliations:** 1 Laboratorio de Bioquímica y Biotecnología Vegetal, Instituto de Hortofruticultura Subtropical y Mediterránea (IHSM), Universidad de Málaga-Consejo Superior de Investigaciones Científicas, Departamento de Biología Molecular y Bioquímica, Facultad de Ciencias, UMA, Málaga, Spain; 2 Sistemas Genómicos, Valencia, Spain

**Keywords:** *AP3*, CRISPR/Cas9, flower development, *Fragaria*, *ananassa*, *Fragaria vesca*, genome editing, MADS-box transcription factor, octoploid, strawberry, *TM6*

## Abstract

The B-class of MADS-box transcription factors has been studied in many plant species, but remains functionally uncharacterized in Rosaceae. APETALA3 (AP3), a member of this class, controls petal and stamen identities in Arabidopsis. In this study, we identified two members of the AP3 lineage in cultivated strawberry, *Fragaria* × *ananassa*, namely *FaAP3* and *FaTM6*. *FaTM6*, and not *FaAP3*, showed an expression pattern equivalent to that of *AP3* in Arabidopsis. We used the CRISPR/Cas9 genome editing system for the first time in an octoploid species to characterize the function of TM6 in strawberry flower development. An analysis by high-throughput sequencing of the *FaTM6* locus spanning the target sites showed highly efficient genome editing already present in the T0 generation. Phenotypic characterization of the mutant lines indicated that FaTM6 plays a key role in anther development in strawberry. Our results validate the use of the CRISPR/Cas9 system for gene functional analysis in *F.* × *ananassa* as an octoploid species, and offer new opportunities for engineering strawberry to improve traits of interest in breeding programs.

## Teaser

CRISPR/Cas9-mediated mutagenesis is used for the first time in an octoploid species, the cultivated strawberry, to functionally characterize the role of the B-class MADS box gene *FaTM6* in flower development

## Introduction

In the years that have passed since the formulation of the classic ABC model for floral organ identity (Coen and Meyerowitz, 1991), our understanding of the molecular mechanisms controlling floral organ development has progressed significantly. The activities of the B-class proteins, APETALA3 (AP3) and PISTILLATA (PI), specify petal and stamen identities when combined with the activities of A- and C-class proteins, respectively (Krizek and Meyerowitz, 1996). *AP3* and *PI* arose from an ancestral duplication event, which it is suggested occurred before the diversification of the angiosperms (Kramer *et al.*, 1998). Later, *AP3* experienced a second duplication before the diversification of the higher eudicots in the *AP3* lineage, resulting in two paralogous lineages, *euAP3* and *Tomato MADS box gene6* (*TM6*), which differ in their C-terminal sequence motifs (Pnueli *et al.*, 1991; Kramer *et al.*, 1998). Although there are species that have lost one of the lineages, such as Arabidopsis and Antirrhinum, which lack *TM6*, and papaya, where *euAP3* is absent (Causier *et al.*, 2010), most species posses both *euAP3* and *TM6* genes, which have functionally diversified. *euAP3* genes, such as the Arabidopsis *AP3*, are mainly involved in both petal and stamen development (Jack *et al.*, 1992). By contrast, *TM6-like* genes have a predominant role in stamens (de Martino *et al.*, 2006; Rijpkema *et al.*, 2006; Roque *et al.*, 2013).

In strawberry (*Fragaria*), flowers differ from those of Arabidopsis in several aspects. The most striking difference is the presence of hundreds of independent carpels located on an enlarged stem tip, the receptacle, which expands upon carpel fertilization to generate the fleshy part of the berry, surrounded by the true fruits, the achenes (Nitsch, 1950; Hollender *et al.*, 2012). A role in the ABC model of flower development has not yet been functionally determined for any of the homeotic genes in strawberry, and it is not yet known whether or how these genes contribute to the development of this particular type of flower.

Strawberry possesses a wide range of ploidy levels, varying from diploid, such as the ancestral species *F. vesca* (woodland strawberry, 2*n*=2*x*=14 chromosomes), to decaploid, such as *F. iturupensis* (2*n*=10*x*=70). The cultivated strawberry, *F.* × *ananassa*, is an octoploid species (2*n*=8*x*=56). Recent studies have proposed that the complex origin of the *F.* × *ananassa* genome was the result of hybridization of three or four different species with different levels of ploidy (Tennessen *et al.*, 2014; Sargent *et al.*, 2016). Recently, a virtual reference genome of *F.* × *ananassa* has been established after sequencing some wild relatives (Hirakawa *et al.*, 2014); however, a whole-genome sequence for this species has not yet been published. As an alternative, the genome of the diploid *F. vesca* is commonly used as the reference (Shulaev *et al.*, 2011; Tennessen *et al.*, 2013, 2014; Li *et al.*, 2017; Edger *et al.*, 2018).

Reverse-genetics strategies employed to characterize gene function in strawberry are based on gene down-regulation via post-transcriptional gene-silencing by RNA interference (RNAi) (Guidarelli and Baraldi, 2015). Although it is an essential tool for gene functional studies, the RNAi approach has two main drawbacks: (1) it requires active repression, which may result in incomplete and/or temporary knockdown effects and therefore to an undesirable background expression of the gene (Barrangou *et al.*, 2015); and (2) it triggers gene-silencing by small interfering RNAs (siRNAs), which can lead to non-specific or off-target effects that are widespread and unpredictable (Birmingham *et al.*, 2014). Recent progress in genome editing methods has opened up new possibilities for reverse-genetics studies. In particular, the Clustered Regularly Interspaced Short Palindromic Repeat (CRISPR)/CRISPR-associated 9 endonuclease (Cas9) technology (hereafter CRISPR/Cas9) has become established as a very powerful tool for the introduction of desired mutations due to its simplicity, efficiency, and stability. CRISPR/Cas9-mediated mutagenesis has been widely applied to plant research in the last few years, not only in Arabidopsis, (Jiang *et al.*, 2013, 2014; Li *et al.*, 2013; Feng *et al.*, 2014; Gao and Zhao, 2014) but also in Rosaceae species, such as apple (Malnoy *et al.*, 2016; Nishitani *et al.*, 2016) and, recently, the diploid wild strawberry *F. vesca* (Zhou *et al.*, 2018). CRISPR/Cas9 has also been used in crops with high ploidy levels such as citrus (triploid), potato, oilseed rape, cotton (tetraploids), and bread wheat (hexaploid) (Weeks, 2017). However, the functionality of this genome editing system has yet to be tested in an octoploid species such as *F.* × *ananassa*.

In this study, we used CRISPR/Cas9 to functionally characterize the role of a homeotic gene, *FaTM6*, in *F.* × *ananassa*. Mutations of this gene affect the development of petals, anthers, and pollen grains, and subsequently of berries. This work demonstrates that FaTM6 plays a role equivalent to AP3 in Arabidopsis, and that the CRISPR/Cas9 system can be a suitable tool for functional analyses and molecular breeding in the cultivated strawberry species, which may have important economic implications.

## Material and methods

### Alignment and phylogenetic tree of AP3- and TM6-like proteins

The *Arabidopsis thaliana* AP3 protein sequence was used as a query in a BLAST search against translated protein sequences of the strawberry genome (v. 4.0.a1) (Edger *et al.*, 2018) in the Genome Database for Rosaceae (https://www.rosaceae.org/) to obtain *Fragaria vesca* TM6 (FveTM6) and AP3 (FveAP3) protein sequences. Multiple sequence alignments of AP3- and TM6-like proteins were performed using MUSCLE with the SeaView version 4 program (Gouy *et al.*, 2010). The phylogenetic tree was inferred by the neighbor-joining method. A total of 1000 bootstrap pseudo-replicates were used to estimate the reliability of internal nodes. Evolutionary distances were computed using the Poisson correction method. The tree was rooted using four PI-like sequences, namely At-PI (*A. thaliana*), TPI (*Solanum lycopersicum*), FvePI-1, and FvePI-2 (*F. vesca*). Tree inference was performed using MEGA version 7 (Kumar *et al.*, 2016). The dataset comprised 68 previously reported *AP3*- and *TM6*-like genes from gymnosperms, monocots, basal angiosperms, basal eudicots, and core eudicots obtained from GenBank.

### Plant material, transient and stable transformation

*Fragaria vesca* (cv. Reine des Vallées) and *F.* × *ananassa* Duch. (cv. Camarosa) plants were grown and maintained under greenhouse conditions (IHSM, Málaga, Spain). Adult *tm6* mutant plants were grown under the same conditions but in an independent and isolated greenhouse module to avoid cross-contamination with wild-type pollen. Transient expression of the sgRNA1-2/Cas9 binary vector was performed by infiltration of a suspension of *Agrobacterium tumefaciens* (strain AGL-0) into fruits at the green stage of development of *F. vesca* as previously described (Hoffmann *et al.*, 2006). Fruits were collected 10 d after the infiltration when they reached the red stage. For stable transformation of *F.* × *ananassa* cv. Camarosa, plants were micropropagated in N30K medium supplemented with 2.20 μM kinetin. Transformation was performed according to the protocol described by Barceló *et al.* (1998). Leaf discs were transformed with *A. tumefaciens* (strain LBA4404) carrying a pCAMBIA2300 plasmid that contained the kanamycin resistance gene *nptII* and the sgRNA1-2/Cas9 cassette. Regenerated shoots were selected in the same medium supplemented with 50 mg l^−1^ kanamycin and 500 mg l^−1^ carbenicillin. Resistant plants were transferred to the greenhouse at 20–30 weeks post-transformation.

### Design of sgRNAs and vector constructs

The genomic sequence of *FveTM6* (FvH4_1g12260), previously annotated as gene14896-v1.0-hybrid, was obtained from the reference genome of Shulaev *et al.* (2011). Single-guide (sg)RNAs were designed using the ATUM CRISPR/gRNA tool (https://www.atum.bio/eCommerce/cas9/input) with *FveTM6* CDS as the input sequence. We performed a BLAST search of *FveTM6* CDS against the *F. vesca* reference genome to select those candidate sgRNAs that were specific to the target gene. Two sgRNAs located in exon 1 and exon 2, and separated by 198 bp (from PAM to PAM) were selected. The CRISPOR web-tool (http://crispor.org) (Haeussler *et al.*, 2016) was used to validate the quality of the selected sgRNAs and to identify putative off-targets.

Two different vectors were used to generate the final sgRNA1-2/Cas9 construct, namely pAtU6:sgRNA and 35S:hSpCas9 (Mao *et al.*, 2013). We cloned both sgRNA1 and sgRNA2 into pAtU6:sgRNA vectors using BbsI. pAtU6:sgRNA1 was cloned into the 35S:hSpCas9 vector using Acc65I and SalI. pAtU6:sgRNA2 was amplified to include the Cfr9 and XbaI restriction sites and cloned into the pAtU6:sgRNA1/35S:hSpCas9 vector, generating a construct with Cas9 and the two sgRNAs cassettes. The final binary vector was obtained by cloning the sgRNA1-2/Cas9 cassette into pCAMBIA2300 using KpnI and XbaI sites. The binary vector was introduced into *A. tumefaciens* strain AGL-0 for transient expression and into LBA4404 for stable transformation.

### Mutation identification

Genomic DNA was isolated using the CTAB method from fruits of the transiently transformed plants, and from leaves of the stably transformed plants. The presence of the transformation cassette was tested by PCR using primers P248 and P249 to amplify Cas9 (Supplementary Table S2 at *JXB* online). CRISPR/Cas9-mediated indels were detected by agarose gel electrophoresis after PCR using primers flanking both sgRNAs (P180/P181 for transient assay, and P445/P446 for stable lines; Supplementary Table S2). For transient expression experiments, the PCR amplicons were cloned into the pGEMT-Easy vector system (Promega, Madison, USA) and transformed into *E. coli* DH5α. Single colonies were picked to identify mutations by Sanger sequencing.

### Amplicon sequencing and sequence analysis

cDNA from petals and stamens was amplified using P445 and P475, and genomic DNA from leaves was amplified using P445 and P446 (Supplementary Table S2) for high-throughput amplicon sequencing. Resulting amplicons were reamplified for indexing. Libraries were purified with Agencourt^®^ AMPure^®^ XP beads (Beckman Coulter) using the manufacturer’s recommendations, and their quality was verified using a TapeStation 4200 HS DNA (Agilent). Libraries were quantified using real-time PCR, and they were pooled in equimolar ratios before paired-end sequencing (2 × 250 cycles) using a MiSeq system (Illumina).

For the sequence analysis, the paired-end reads were collapsed using the FLASH algorithm developed by Magoč and Salzberg (2011), using a Phred quality score of 33 as a filter. The resulting clusters were then transformed to the FASTA format using a custom Python script. In order to reduce noise, 35 nucleotides in the 5′ and 3′ positions were trimmed using Trimmomatic (Bolger *et al.*, 2014). Custom Python scripts were designed for sequence identification and quantification. For the quantification process, no similarity threshold for sequence clusters was applied. Sequences with at least 1% prevalence were selected as possible allelic variants. However, PCR bias resulted in some missing alleles. In these rare cases, a fingerprint strategy based on SNPs exclusive for the missing allele, allowing variations among the target sites, was designed using custom Python scripts.

The sequences of two putative off-targets located within the coding sequences (off-targets #1 and #3 of the sgRNA1; Supplementary Table S1) were analysed by amplifying the spanning region of both loci with the oligonucleotides P546/P547 and P544/P545, respectively (Supplementary Table S2). The resulting PCR products were cloned into pGEM-T Easy vectors (Promega). For each line, 40 and 45 clones were selected from the constructs for off-targets #1 and #3, respectively, and these were analysed by Sanger sequencing.

### Phenotypic analyses

For control plants and *tm6* mutant lines, flowers at the pre-anthesis stage were marked so that all phenotypic analyses could be performed at the same developmental stage. SEM visualization of stamens was performed using flowers at 2 d post-anthesis, and that of carpels at the pre-anthesis stage. Stamens and carpels were visualized without processing using a JEOL JSM-6490LV electron microscope under low-vacuum conditions (30 MPa). To examine the pollen morphology, anthers at the dehiscence stage were incubated in absolute ethanol for 3 h, air-dried, and coated with gold in a sputtering Quorum Q150R ES. Gold-coated pollen grains were examined using a MEB JEOL 840 microscope.

### Quantification and germination of pollen grains

To quantify the pollen content and analyse their germination in control and *tm6* mutant lines, three flowers per genotype were collected at 2 d post-anthesis. For the quantification assay, anthers were removed and incubated with 10% sucrose and 1% acetocarmine, which stains nuclei and is therefore used as a marker for pollen viability (Chang and Neuffer, 1989). Pollen grains were quantified using a Neubauer chamber under a stereomicroscope Multizoom AZ-100 (Nikon). The pollen germination assay was performed as previously described by Ariza *et al.* (2006). The samples were visualized under Eclipse E800 microscope (Nikon).

### Emasculation and cross-pollination

Emasculation was performed by removing all of the stamens in control flowers at the pre-anthesis stage. In order to avoid cross-pollination, the emasculated flowers were covered with cotton. To analyse the functionality of the carpels in *tm6* lines, *tm6-7* flowers were pollinated using wild-type pollen. Briefly, the anthers of the mutant flowers were removed manually at pre-anthesis to avoid any possible self-fertilization. A small paintbrush was then used to pollinate the *tm6* stigmas with wild-type pollen. In order to avoid cross-contamination, cross-pollinated flowers were covered with cotton.

### Accession numbers

Most of the protein sequences were obtained from GenBank: MASAKO B3 (*Rosa rugosa*; AB055966), PaTM6 (*Prunus avium*; AB763909), MdMADS13 (*Malus* × *domestica*; AJ251116), MdTM6 (*M.* × *domestica*; AB081093), HmTM6 (*Hydrangea macrophylla*; AF230703), GDEF1 (*Gerbera hybrida*; AJ009724), VvTM6 (*Vitis vinifera*; DQ979341), BalTM6 (*Balanophora fungosa*; JQ613232), PhTM6 (*Petunia* × *hybrida*; DQ539417), LeTM6 (*Solanum lycopersicum*; X60759), NbTM6 (*Nicotiana benthamiana*; AY577817), PtAP3-2 (*Pachysandra terminalis*; AF052871), PtAP3-1 (*P. terminalis*; AF052870), Gu.ti. AP3-5 (*Gunnera tinctoria*; AY337757), Gu.ti. AP3-4 (*G. tinctoria*; AY337756), FavAP31.1 (*Fragaria* × *ananassa*; AY429427), MASAKO euB3 (*R. rugosa*; AB099875), GDEF2 (*G. hybrida*; AJ009725), HpDEF2 (*Hieracium piloselloides*; AF180365), HpDEF1 (*H. piloselloides*; AF180364), AtAP3 (*Arabidopsis thaliana;*AF115814), CMB2 (*Dianthus caryophyllus*; L40405), SLM3 (*Silene latifolia*; X80490), RAD2 (*Rumex acetosa*; X89108), RAD1 (*R. acetosa*; X89113), JrAP3 (*Juglans regia*; AJ313089), RfAP3-2 (*Ranunculus ficaria*; AF130870), RfAP3-1 (*R. ficaria*; AF052854), HmAP3 (*H. macrophylla*; AF230702), NMH7 (*Medicago sativa*; L41727), VvAP3 (*V. vinifera*; EF418603), CitMADS8 (*Citrus unshui*; AB218614), DEF (*Antirrhinum majus*; X52023), NtDEF (*Nicotiana tabacum*; X96428), PhDEF (*P.* × *hybrida*; DQ539416), StDEF (*Solanum tuberosum*; X67511), TAP3 (*S. lycopersicum*; DQ674532), LeAP3 (*S. lycopersicum*; AF052868), RfAP3-1 (*R. ficaria*; AF052854), RbAP3-1 (*Ranunculus bulbosus*; AF052876), RfAP3-2 (*R. ficaria*; AF130870), RbAP3-2 (*R. bulbosus*; AF130869), PnAP3-1 (*Papaver nudiculae*; AF052873), PapsAP3-1 (*Papaver somniferum*; EF071993), PcAP3 (*Papaver californicum*; AF052872), LtAP3 (*Liriodendron tulipifera*; AF052878), MpMADS7 (*Magnolia praecocissima*; AB050649), Pe.am.AP3 (*Persea americana*; AY337748), CfAP3-1 (*Calycanthus floridus*; AF230699), CfAP3-2 (*C. floridus*; AF230700), PeMADS2 (*Phalaenopsis equestris*; AY378149), PeMADS5 (*P. equestris*; AY378148), OsMADS16 (*Oryza sativa*; AF077760), SILKY1 (*Zea mays*; AF181479), PeMADS4 (*P. equestris*; AY378147), LMADS1 (*Lilium longiflorum*; AF503913), LRDEF (*Lilium regale*; AB071378), CryMADS1 (*Cryptomeria japonica*; AF097746), CryMADS2 (*C. japonica*; AF097747), GnegGGM2 (*Gnetum gnemon*; AJ132208), GnegGGM13 (*G. gnemon*; AJ132219), DAL13-1 (*Picea abies*; AF158543), PrDGL (*Pinus radiata*; AF120097), GnegGGM15 (*G. gnemon*; AJ251555), TPI (*S. lycopersicum*; DQ674531)

FveTM6 (*Fragaria vesca*; FvH4_1g12260), FveAP3 (*F. vesca*; FvH4_2g38970). AtPI (*A. thaliana*; At5g20240), FvePI-1 (*F. vesca*; FvH4_2g27860.1), FvePI-2 (*F. vesca*; FvH4_2g278270.1).

## Results

### Identification and phylogenetic analysis of *AP3* lineage genes in *F. vesca*

To identify genes belonging to the *AP3* lineage in strawberry, a BLAST search was performed using the AP3 protein sequence from Arabidopsis (AtAP3) against the reference genome of the diploid *F. vesca* (cv. Hawaii 4). Two genes with high homology were obtained, FvH4_2g38970 and FvH4_1g12260, sharing 55.45% and 50.43% of amino acid identity with AtAP3, respectively. To place these two genes in a phylogenetic context, we performed a phylogenetic analysis using neighbor-joining of AP3- and TM6-like proteins from gymnosperms to core eudicots (Supplementary Fig. S1). The phylogenetic analysis showed that FvH4_2g38970 (hereafter named FveAP3) and FvH4_1g12260 (hereafter named FveTM6) belong to the *euAP3* and *TM6* lineages, respectively, indicating that *F. vesca*, unlike Arabidopsis, contains both AP3 lineages.

### Expression analysis of *AP3* lineage genes in *F.* × *ananassa*

**Fig. 1.**
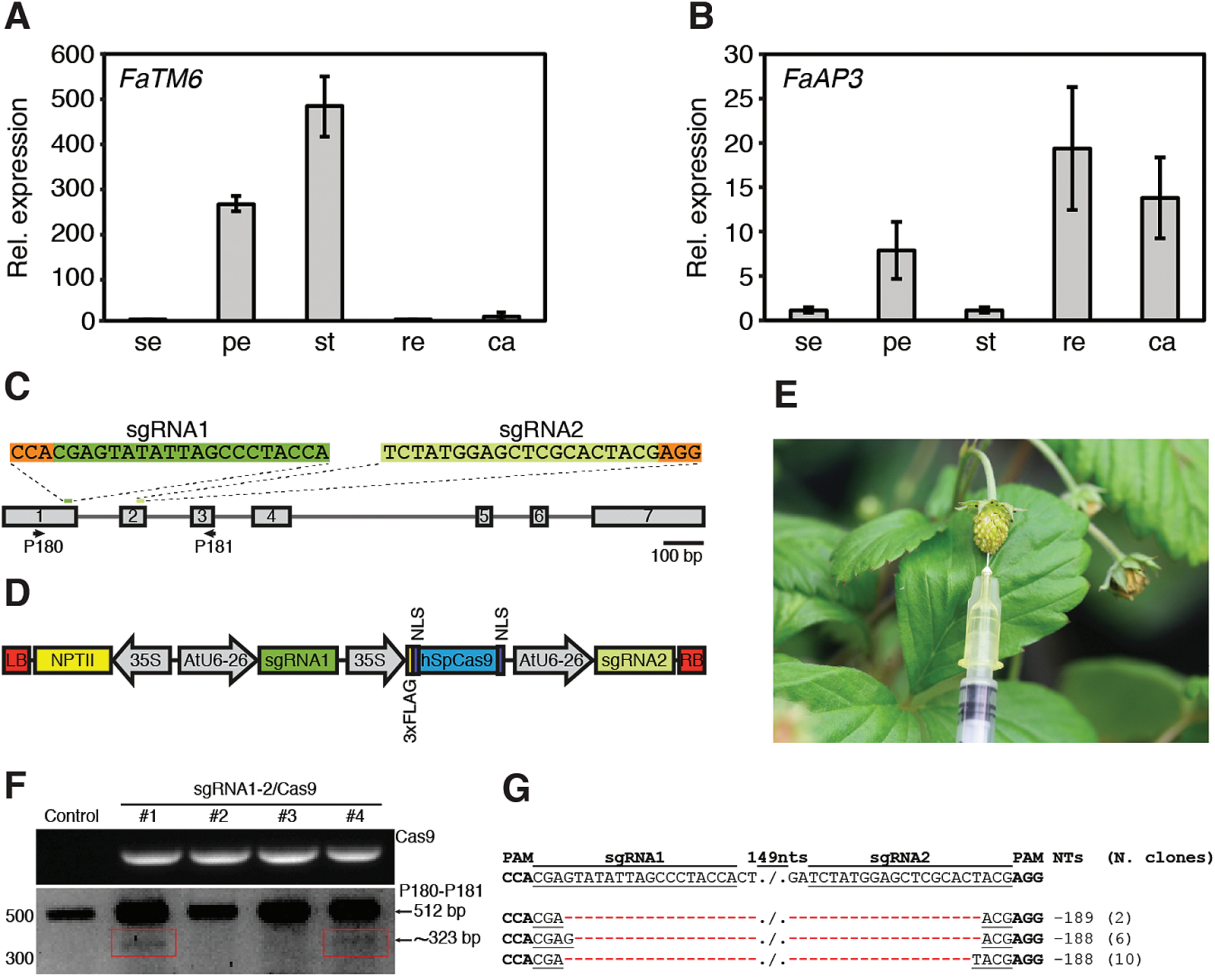
Expression of *AP3* and *TM6* genes in the cultivated strawberry (*Fragaria* × *ananassa*), construct design, and evaluation of CRISPR-Cas9-based editing of the wild strawberry, *Fragaria vesca*. Relative expression of the *F.* × *ananassa TM6* gene (*FaTM6*) (A) and *AP3* gene (*FaAP3*) (B) in sepals (se), petals (pe), stamens (st), receptacles (re), and carpels (ca) of *F.* × *ananassa* flowers at stage 12 as determined by qRT-PCR. Data are means (±SD) of three biological replicates with three technical replicates each. (C) The *F. vesca TM6* (*FveTM6*) locus, including exons (boxes) and introns (lines). sgRNAs 1 and 2 are represented in green and PAM sequences in orange. The primers used for characterization of CRISPR/Cas9 editing are indicated. (D) Schematic representation of the two sgRNAs and Cas9 expression cassettes in a single binary vector pCMABIA2300 (sgRNA1-2/Cas9 vector). (E) Green fruit agro-infiltrated with the sgRNA1-2/Cas9 vector. (F) PCR to detect indels in *FveTM6*. Top panel: PCR of Cas9 in four fruits that transiently expressed the vector. Bottom panel: PCR with P180 and P181 (Supplementary Table S2) showing a ~323-bp band (red boxes) in fruits #1 and #4 in addition to the wild-type band (512 bp). (G) Alignment of sequences obtained after the purification, cloning, and Sanger sequencing of the ~323 bp band from fruits #1 and #4. Fragments with 189- and 188-bp deletions resulting from simultaneous DSBs in both target sites were detected in two and 16 clones, respectively.

To further investigate the role of the *AP3* lineage genes in cultivated strawberry, we first analysed their expression using quantitative real-time PCR (qRT-PCR) in sepals, petals, stamens, receptacles, and carpels of *F.* × *ananassa* flowers at stage 12 (Hollender *et al.*, 2012). As shown in Fig. 1A, *FaTM6* was expressed in both petals and stamens, with the latter having the higher expression level. In contrast, *FaAP3* was expressed mainly in receptacles, followed by carpels and petals, with very little expression in stamens and sepals (Fig. 1B). This suggested that *FaTM6*, and not *FaAP3*, is the gene with homologous function to *AP3* in Arabidopsis. Hence, we selected *FaTM6* to study its role in flower development by CRISPR/Cas9-mediated mutagenesis.

### Identification of *FaTM6* targets, characterization of *FaTM6* alleles, and construct design

We first searched for candidate sgRNAs to edit *FaTM6* using the available sequence of *FveTM6* from the *F. vesca* reference genome (cv. Hawaii 4). Two target sites for *FveTM6* were selected in order to generate a dual sgRNA construct, allowing us to create a large deletion and/or increase the efficiency of the mutagenesis (Belhaj *et al.*, 2013). The sgRNAs, located within exon 1 (sgRNA1) and exon 2 (sgRNA2), were selected based on their high specificity score and minimum potential off-target activities (Fig. 1C, Supplementary Table S1). sgRNA1 spanned the carboxyl-end of the M-domain and the amino-terminal of the ‘intervening’ (I) region of the FveTM6 protein, while sgRNA2 was located within the I region (Supplementary Fig. S2).

Next, we evaluated the suitability of the two guides designed using the reference genome for editing the two genotypes used in this study [*F. vesca* cv. Reine des Vallées (RV) and *F.* × *ananassa* cv. Camarosa]. In order to detect any polymorphisms in the reference genome that might affect the CRISPR/Cas9-mediated editing, we amplified and cloned the genomic regions of *TM6* spanning the two target sites (Fig. 1C, Supplementary Table S2) and performed Sanger sequencing. While *F. vesca* cv. RV did not show any variation in the *TM6* sequence compared to the reference genome, five different alleles were identified in *F.* × *ananassa* cv. Camarosa (Supplementary Fig. S3). These alleles contained indels in the first and second introns, and four synonymous and eight non-synonymous SNPs within the coding region. None of these alleles had polymorphisms in the region targeted by sgRNA2, but allele #5 contained a G168T substitution within the PAM-proximal region of sgRNA1 (Supplementary Fig. S3), which might decrease the cleavage efficiency (Xu *et al.*, 2017). To determine which alleles were expressed in petals and stamens, we generated cDNA from these tissues and performed high-throughput amplicon sequencing for the region spanning sgRNA1 and sgRNA2. Our data indicated that at least four of the five *FaTM6* alleles identified were expressed in both petals and stamens (Supplementary Table S3). Specifically, we detected alleles #3, #4, #5, and a sequence that might correspond to either allele #1 or #2, which are indistinguishable within the sequenced CDS region. Given this information and with our dual sgRNA strategy, sgRNA2 would probably ensure editing events due to the lack of polymorphisms, while sgRNA1 would allow us to assess the effect of the mismatch in allele #5 on editing efficiency. This design would also result in large deletions if both sites were simultaneously cleaved by Cas9, probably producing non-functional alleles. We designed a single binary vector harboring the two sgRNAs under AtU6-26 promoters, and the Cas9 nuclease under the 35SCaMV promoter, sgRNA1-2/Cas9 (Fig. 1D).

### Functionality test of the sgRNA1-2/Cas9 vector by transient transformation of *F. vesca* fruits

Since the transformation and establishment of stable transgenic plants requires 6–9 months, we first tested the functionality of our dual sgRNA/Cas9 editing construct by transient transformation of diploid *F. vesca* cv. RV fruits. The sgRNA1-2/Cas9 vector was agro-infiltrated in the receptacle of fruits at the green stage (Fig. 1E), and genomic DNA was extracted at 10 d post-infiltration. PCR amplification was performed with primers spanning the target sites (Supplementary Table S2) in order to detect CRISPR/Cas9-mediated editing events as evidenced by amplicon sizes that were different from the wild-type allele (Fig. 1C). The PCR results confirmed that two of the four fruits infiltrated with the sgRNA1-2/Cas9 vector showed a smaller amplicon in addition to the wild-type (Fig. 1F). Cloning and Sanger sequencing of the smaller amplicon from these two plants confirmed the presence of a deletion of ~190 bp between the two target sites in 18 clones, validating the functionality of the sgRNA1-2/Cas9 vector in the diploid strawberry species (Fig. 1G).

### Targeted mutagenesis of *FaTM6* in stable transgenic *F.* × *ananassa* plants

**Fig. 2.**
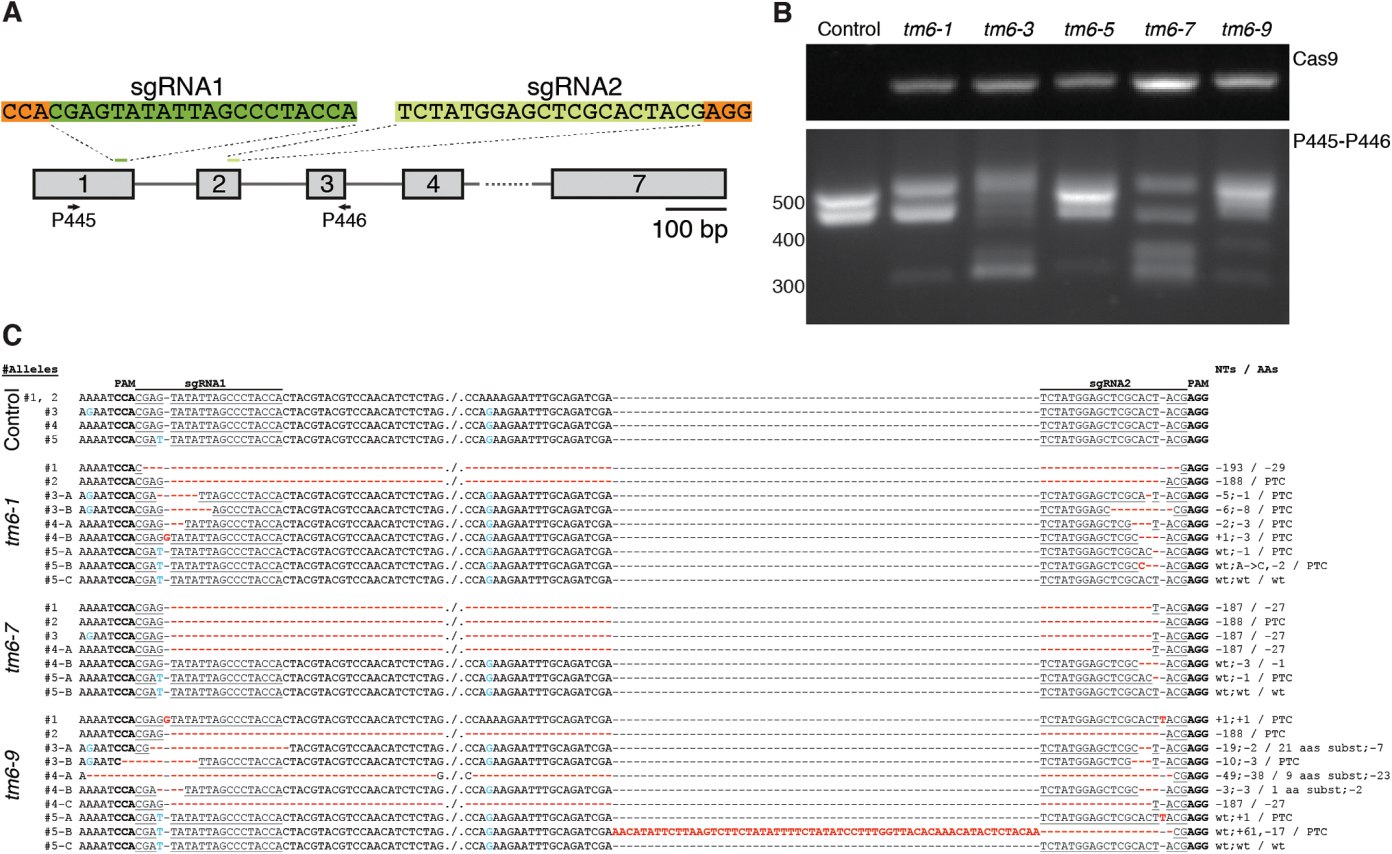
Identification of CRISPR/Cas9-induced mutations in the *Fragaria* × *ananassa TM6* allele (*FaTM6*). (A) Schematic representation of the positions of the sgRNAs in the *F. vesca TM6* (*FveTM6*) locus. The primers used for the analysis in agarose gels and for deep sequencing are represented (P445 and P446; Supplementary Table S2). (B) Top panel: identification of the *Cas9* gene in transgenic *tm6* lines. Bottom panel: detection of mutations at the *FaTM6* locus using the P445 and P446 primers. (C) Sequence alignment obtained by high-throughput amplicon sequencing in control and *tm6* mutant lines. PAM sequences are marked in bold; sgRNAs are underlined; blue indicates distinctive SNPs among *FaTM6* alleles; bold red indicates mutations induced by CRISPR/Cas9-mediated editing. PTC, premature termination codon.

We generated stable transgenic plants of *F.* × *ananassa* cv. Camarosa with the same sgRNA1-2/Cas9 vector. We obtained and micropropagated five independent lines, (*tm6* lines), and used PCR to examine them for editing events, as described for the transient assays (Fig. 2A; Supplementary Table S2). Different amplicon patterns were obtained between the *tm6* lines and the untransformed wild-type plant, indicating the generation of various indels by the CRISPR/Cas9 complex (Fig. 2B). These results confirmed the CRISPR/Cas9-mediated mutagenesis of *FaTM6* in *F.* × *ananassa*.

In order to analyse the CRISPR/Cas9-induced mutations at the *FaTM6* locus at the molecular level, we selected three transgenic lines, *tm6-1*, *tm6-7*, and *tm6-9*, based on their amplicon patterns. Using high-throughput amplicon sequencing and *de novo* assembly, nine, seven, and 10 different alignment groups were obtained for *tm6-1*, *tm6-7*, and *tm6-9*, respectively (Fig. 2C; Supplementary Table S3). Allele #1 showed a single editing event in all three lines, while allele #2 had the same deletion in all of them. However, different editing events were obtained for alleles #3, #4, and #5 within each transgenic line, except for *tm6-7*, which had only one modification in allele #3 (Fig. 2C). As expected, most of the CRISPR/Cas9-induced mutations occurred downstream of the PAM sequences, although a deletion including the whole PAM-sgRNA1 region was also observed in *tm6-9*.

All sequences obtained for alleles #1 through to #4 showed mutations within the region targeted by sgRNA1 (Fig. 2C). However, no editing was observed in this target site in allele #5, most likely due to the mismatch present in the sgRNA1 seed sequence. For the region targeted by sgRNA2, the sequencing analysis showed that all five alleles were edited, although wild-type sequences were also detected for this target, but only in allele #5.

As expected from the amplicon pattern obtained for these three lines, all of them contained large deletions generated by the simultaneous double-strand breaks (DSBs) in both target sites. These deletions of 187 and 193 nts resulted in a deletion of 27 or 29 amino acids, respectively, or in the generation of a premature stop codon (–188 nts) (Fig. 2C, Supplementary Fig. S4). Most of the editing events generated frameshift mutations, especially in the *tm6-1* line, which resulted in the production of seven truncated proteins (#2, #3A, #3B, #4A, #4B, #5A, and #5B) out of the nine allelic variants detected (Supplementary Fig. S4). In addition to the generation of these truncated proteins, shorter amino acid deletions and substitutions were also obtained in *tm6-7* and *tm6-9*.

### Off-target analysis

Seven putative off-targets were predicted for sgRNA1 and sgRNA2 (Supplementary Table 1) using the CRISPOR web tool (http://crispor.org) (Haeussler *et al.*, 2016). Out of these putative off-targets, only #1 and #3 were located at CDSs, containing four and five mismatches within the sgRNA1-PAM sequence, respectively. On the other hand, these two genes were very weakly expressed in petals and anthers of *F. vesca* (Supplementary Fig. S5; Hawkins *et al.*, 2017). Thus, we did not expect any homeotic effect due to possible off-target activities affecting these two genes. Nevertheless, to confirm this hypothesis, we amplified and sequenced the spanning region of both off-targets in the wild-type and *tm6-9* mutant lines. As expected, we did not find any sequence variations in the off-target sequences of *tm6-9* compared to that of the control (Supplementary Fig. S6).

### FaTM6 plays a key role in anther development

**Fig. 3.**
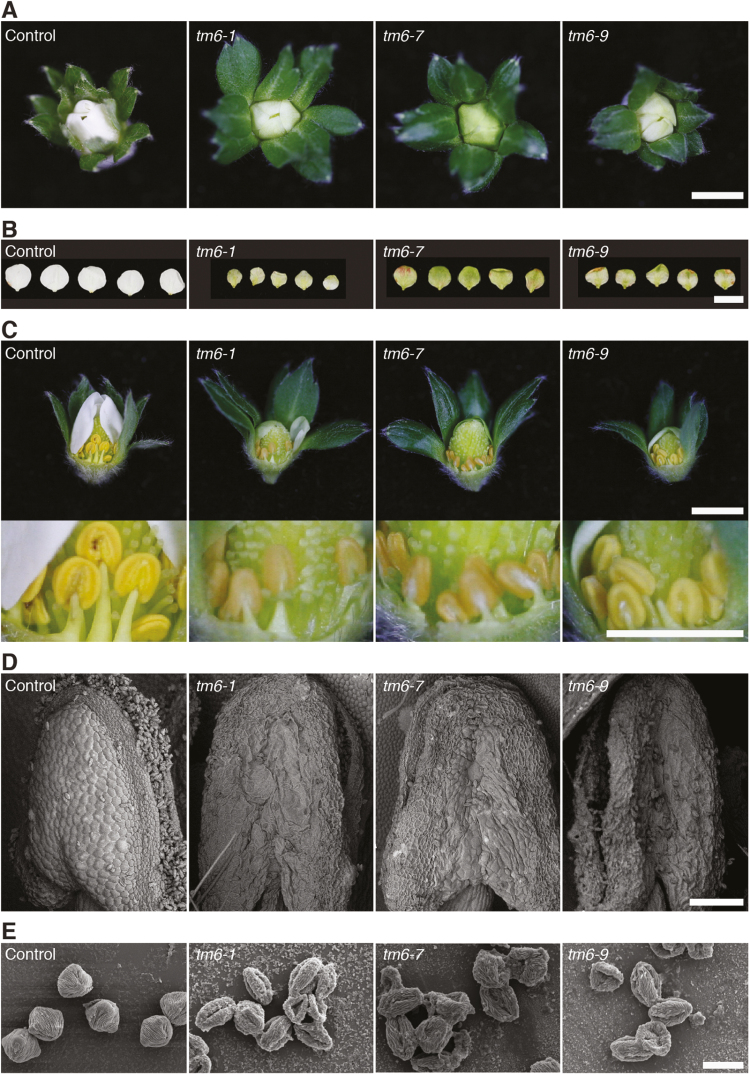
Phenotypic effects of mutations in *Fragaria* × *ananassa TM6* (*FaTM6*) in flowers. (A) Flowers of control and three independent *tm6* lines at the pre-anthesis stage. (B) Petals of *tm6* lines appear smaller and greener. (C) Top panel: flowers at pre-anthesis with some petals removed. Bottom panel: higher magnification to show details of the morphology of the stamens. (D, E), SEM of the structures of the anthers at the dehiscence stage (D) and of the pollen grains (E). Scale bars: (A–C) 1 cm; (D) 200 µm; (E) 20 µm.

To determine the function of *FaTM6*, we analysed the flower phenotype of the wild-type and the *tm6-1*, *tm6-7*, and *tm6-9* mutant lines. At the pre-anthesis stage, petals in the mutant lines were shorter and slightly greener compared to those of the control flowers (Fig. 3A, B). More severe defects were observed in the anthers, which were smaller and darker than that of the wild-type (Fig. 3C). A more detailed anatomical analysis of the anthers at the dehiscence stage and the pollen grains was performed using SEM. Wild-type anthers displayed the typical four-lobed structure, with a very well-defined epidermal layer, and with pollen grains visible at the stomium rupture site (Fig. 3D). However, the anthers of the *tm6* mutant lines displayed morphological differences, showing clear defects in the epidermal cell layer and a reduced number of pollen grains at the stomium. This apparent difference in pollen content was quantified as a 10-fold reduction in *tm6-1* and *tm6-7*, and a 50-fold reduction in *tm6-9* compared to the control (Supplementary Fig. S7A, B). Moreover, most of the pollen grains from the mutant lines showed aberrant and collapsed structures (Fig. 3E). To test pollen grain viability, acetocarmine staining and pollen germination assays were performed. While acetocarmine successfully stained nuclei in the mutant pollen grains (Supplementary Fig. S7A), we failed to detect any germination in the *tm6* mutant lines, in contrast to the wild-type (Supplementary Fig. S7C), indicating that the viability had been strongly impaired in the mutant lines.

**Fig. 4.**
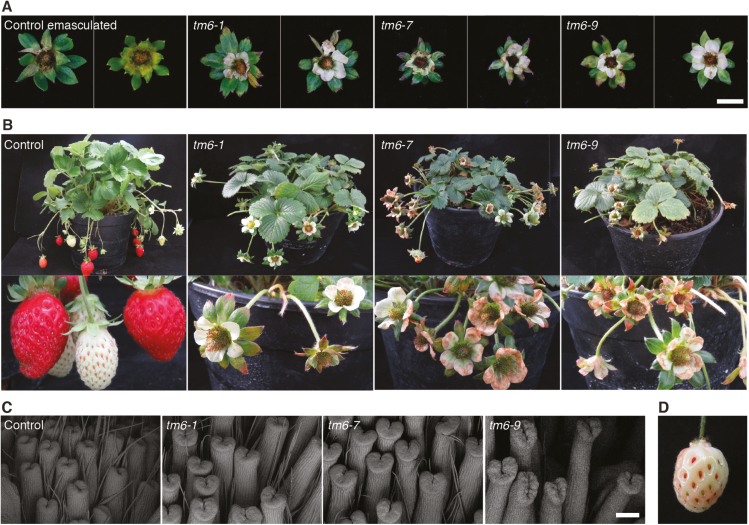
Phenotypic effects of mutations in *Fragaria* × *ananassa TM6* (*FaTM6*) in fruits and in a complementation experiment. (A) Wild-type flowers emasculated at the pre-anthesis stage phenocopy aborted flowers in *tm6* mutant lines. Two representative flowers per genotype are shown. (B) Top panels: adult plants of control and *tm6* mutant lines. Bottom panels: control plants develop wild-type berries, but *tm6* flowers abort. (C) SEM of the structures of carpels at the pre-anthesis stage. (D) A fruit developed from a *tm6-7* flower emasculated and pollinated with wild-type pollen. Scale bars: (A) 1 cm; (C) 200 µm.

Since ovule fertilization and proper development of embryos and achenes are necessary for normal receptacle development (Nitsch, 1950), we assessed fruit formation in the *tm6* lines compared with emasculated controls. The emasculation of wild-type flowers caused a complete abortion in receptacle development (Fig. 4A). Consistent with the impaired pollen grain formation, the *tm6* mutant lines also showed arrested development of the receptacles (Fig. 4A, B). Nevertheless, a few fruits (5.7 and 2.2% in *tm6-7* and *tm6-9*, respectively) showed a local enlargement of the receptacle around some achenes, indicating that some pollen grains must have retained their viability, allowing a residual pollination (Supplementary Fig. S8).

Consistent with the lack of *FaTM6* expression in carpels, these organs showed normal development (Fig. 4C). In order to confirm the carpel viability in the mutant lines, we pollinated carpels of the *tm6-7* line using wild-type pollen. As shown in Fig. 4D a wild-type receptacle was fully developed, indicating that lack of pollination was responsible for the aborted fruit phenotype in the *tm6* lines. These results taken together indicate an essential role of FaTM6 in anther and pollen formation during flower development of *F.* × *ananassa*.

## Discussion

### FaTM6 is involved in petal and stamen development

Here, we report that strawberry, unlike Arabidopsis, has maintained both an *euAP3* and a *TM6* gene. While FveAP3 contains the euAP3 motif in the C-terminal domain of the protein, FveTM6 possesses a motif more similar to those of the paleoAP3 genes (Supplementary Fig. S2) (Kramer *et al.*, 1998). A transcriptome study during floral development of *F. vesca* reported that *FveTM6* (gene14896-v1.0-hybrid) is strongly expressed in anthers (Hollender *et al.*, 2014), consistent with our results in the octoploid strawberry. However, in that study, *FveTM6* was mis-annotated as *FveAP3*, since our phylogenetic analysis showed that this gene belongs instead to the *TM6* lineage (Supplementary Fig. S1). Moreover, expression analysis of *FaAP3* and *FaTM6* in flowers of *F.* × *ananassa* showed that *FaTM6* and not *FaAP3* was the gene with the typical expression pattern of the B-class type (Fig. 1A, B), which is consistent with previous studies in Rosaceae (Hibino *et al.*, 2006).

Sequencing analyses of the region spanning the CRISPR target sites for *TM6* indicated that the diploid *F. vesca* cultivar used in this study was homozygous at this locus (Supplementary Fig. S3). However, the octoploid strawberry *F.* × *ananassa* cv. Camarosa showed high heterozygosity at the *FaTM6* locus, consistent with the genetically complex genome of this species. At least five alleles of *FaTM6* among the four pairs of homoeologous chromosomes were detected. However, we cannot discount an even more complex scenario involving more *FaTM6* alleles due to the possible presence of additional polymorphisms outside the region covered in this study. Furthermore, deep sequencing of cDNA from petals and sepals showed that at least four of the five *FaTM6* were expressed in both organs, with expression of alleles #3, #4, and #5 confirmed, and one of either allele #1 or allele #2, since these are indistinguishable within the sequenced CDS region (Supplementary Table S3).

The role of *euAP3* genes in petal and stamen specification has been well established (Jack *et al.*, 1992; Schwarz-Sommer *et al.*, 1992; de Martino *et al.*, 2006; Roque *et al.*, 2013). However, there have been fewer reports on the role of TM6-like transcription factors, which play a more important role in stamen identity (Rijpkema *et al.*, 2006; Roque *et al.*, 2013). Our results are also consistent with a predominant role for FaTM6 in stamen development since the anthers in the *tm6* mutant lines were severely affected, and they showed a drastic reduction in pollen content and viability (Fig. 3C–E, Supplementary Fig. S7), while the petals of the *tm6* mutants showed more modest defects in overall size and color (Fig. 3B). This is consistent with the phenotype reported in TM6i (RNAi) lines of tomato, which develop smaller petals that are attributed to a decrease in cell proliferation (de Martino *et al.*, 2006). Similarly, *PTD*, the *Populus trichocarpa TM6* ortholog, has been postulated to play a role in regulating cell proliferation (Sheppard *et al.*, 2000)

Auxin transport from fertilized carpels to the floral receptacle is essential for the latter to grow into a fleshy and edible fruit (Nitsch, 1950; Kang *et al.*, 2013). The high percentage of fruit abortions observed in the *tm6* mutant lines, which phenocopy emasculated wild-type flowers, supports the tight coupling of flower and fruit development (Supplementary Fig. S8, Fig. 4A, B). The presence of a small number of aberrant fruits with enlarged portions of receptacle around developed achenes (Supplementary Fig. S8) indicated that some residual viable pollen was formed despite our failure to detect pollen germination (Supplementary Fig. S7C). *tm6* mutants developed anatomically normal pistils (Fig. 4C), consistent with the lack of *FaTM6* expression in this organ (Fig. 1A). In fact, carpels in the *tm6* lines were functional since fruit development was restored using wild-type pollen. All of these findings indicate that the defect in pollen formation rather than gynoecium development caused the fruit abortions in the *tm6* lines.

### CRISPR/Cas9 is an efficient tool for gene functional analysis in octoploid strawberry

In this study, we designed a dual sgRNA system that efficiently edited the *FaTM6* gene in octoploid strawberry. Moreover, we developed a quick and easy validation *in vivo* of the sgRNA efficiency by performing a transient assay in fruits of the diploid *F. vesca*.

A deep sequence analysis in three independent transgenic CRISPR lines in *F.* × *ananassa* showed a high efficiency of sgRNA1, which drove the editing of four of the five *FaTM6* alleles in all lines examined (Fig. 2C, Supplementary Table S3). Only allele #5 remained totally unedited at this target site, probably because of the mismatch in the seed sequence, which has been reported to decrease the cleavage efficiency (Xu *et al.*, 2017). Although Cas9 can still tolerate mismatches within the target site (Hsu *et al.*, 2013; Braatz *et al.*, 2017), our results indicated that the mismatch in the seed sequence of allele #5 totally prevented the Cas9 activity (Fig. 2C, Supplementary Table S3). None of the alleles had mismatches with sgRNA2, and this led to the editing of all the five *FaTM6* alleles. Interestingly, an unedited variant for allele #5 was also detected with a low prevalence in each line (#5-C, #5-B, and #5-C in *tm6-1*, *tm6-7*, and *tm6-9* respectively; Supplementary Table S3). It is possible that the SNP present at the sgRNA1 target site for allele #5 also affected editing at the sgRNA2 site. All of these results show that a preliminary sequence analysis is essential to optimize the efficiency of the CRISPR/Cas9 system in a polyploid and highly heterozygous species such as *F.* × *ananassa*, especially when a reference genome sequence is not available. Moreover, when designing CRISPR/Cas9 experiments to edit a polymorphic locus of *F.* × *ananassa*, we recommend designing constructs containing multiple sgRNAs against the different allelic variants.

Importantly, and as expected by their number of mismatches to the *TM6* locus, no editing was found in the two putative off-target sites located within CDSs (Supplementary Fig. S6). This result provides strong support for the *tm6* mutations being responsible for the aberrant development of anthers and petals in these mutant lines.

Despite the octoploid nature of strawberry, our analysis showed that the *tm6-1* and *tm6-9* lines contained more than eight allelic variants, indicating that these two lines are chimeras. These genetic mosaics are common in CRISPR/Cas9 T0 generations of plants obtained from somatic tissue due to the activity of Cas9 during later stages of shoot development (Liu *et al.*, 2017). Due to the high heterozygosity of cultivated strawberry, breeding lines can only be maintained and propagated clonally by runners. Therefore, even though a full knock-out was not achieved in the T0 generation, continuous clonal propagation of the transgenic lines containing the CRISPR/Cas9 may enable the eventual mutation of all eight homeologs.

### Conclusions

In summary, we have characterized the role of a homeotic gene in strawberry, *FaTM6*, for the first time. We have shown that it is primarily involved in anther development, but also has a role in petal formation. In obtaining these results, we have demonstrated that CRISPR/Cas9 is a powerful tool for gene functional studies in commercial strawberry that can overcome the drawbacks of RNAi-based approaches, such as incomplete and/or unstable knockdown effects and unpredictable off-targets. Moreover, by confirming the feasibility of genome-editing in *F.* × *ananassa*, we have shown the potential of this approach for future generation of lines with traits of agronomic interest, despite the high ploidy of this species.

## Supplementary data

Supplementary data are available at *JXB* online.

Fig. S1. Phylogenetic analysis of TM6 and euAP3 lineage proteins.

Fig. S2. Alignment of AP3- and TM6-like proteins.

Fig. S3. Alignment of *TM6* sequences from *F. vesca* and *F.* × *ananassa*.

Fig. S4. Alignment of TM6 predicted amino acid sequences in the *tm6* mutant lines.

Fig. S5. Expression analysis of two putative off-targets for sgRNA1.

Fig. S6. Sequence analyses of two putative off-targets for sgRNA1.

Fig. S7. Pollen yield quantification in the *tm6* mutant lines.

Fig. S8. Quantification of the *tm6* fruit phenotype.

Table S1. Off-target analysis for sgRNA1 and sgRNA2.

Table S2. List of oligonucleotides used in this study.

Table S3. Analysis of high-throughput sequencing of amplicons of *TM6* cDNA and genomic DNA.

Supplementary Figure

Supplementary Figure Legends

Supplementary Table
