# Supplementary material for: Functional analysis of the TM6 MADS-box gene in the octoploid strawberry by CRISPR/Cas9-directed mutagenesis
Source: J Exp Bot. 2018 Nov 13;70(3):885–95. doi: 10.1093/jxb/ery400 (PMC6363087; doi:10.1093/jxb/ery400)
Supplement: Supplementary Figure [file ery400_suppl_supplementary_figure.pdf]

Supplemental Figure 1

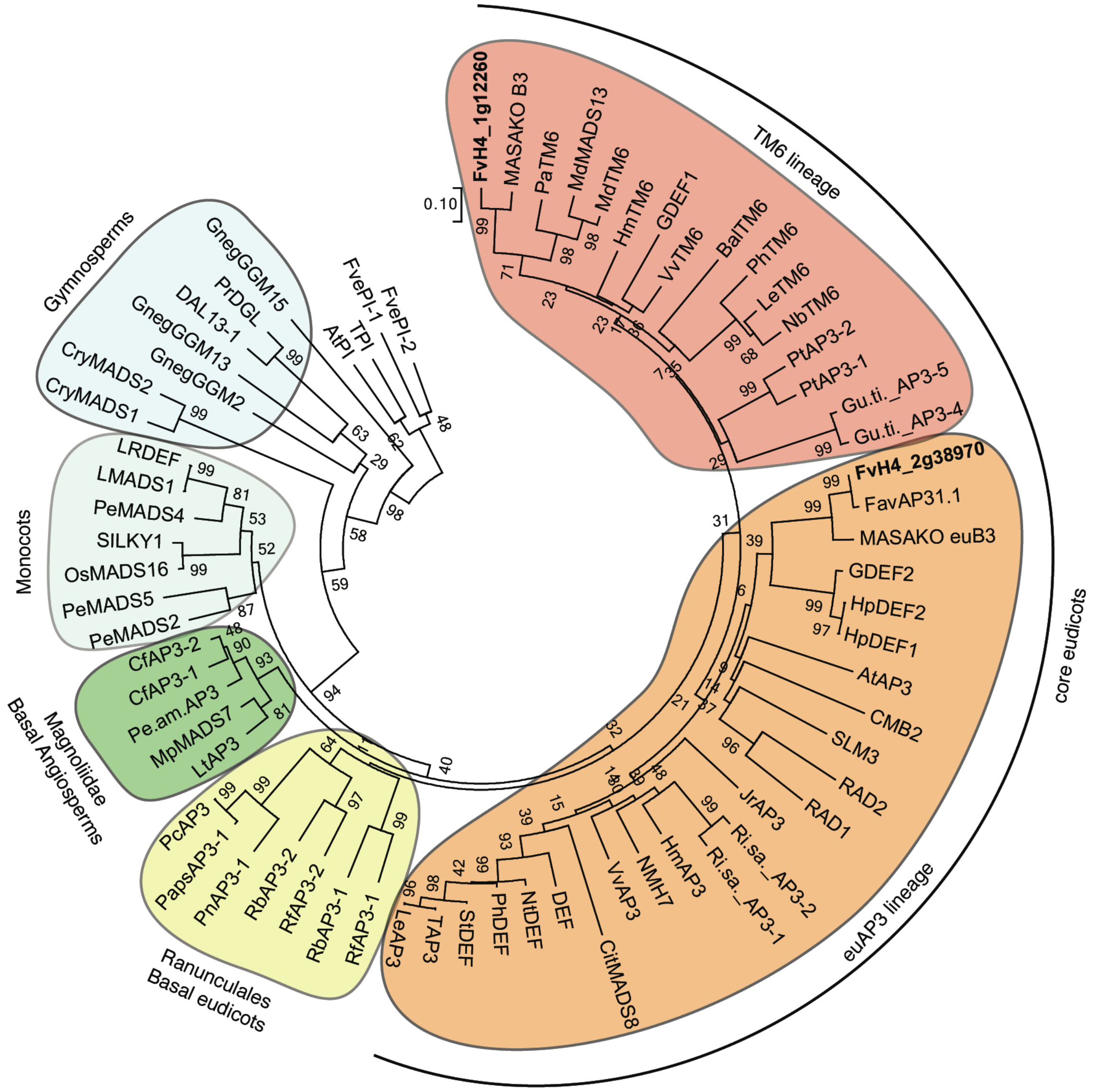

Supplementary Figure S1. Neighbor-Joining Analysis of TM6 and euAP3 lineage proteins. Representative AP3 lineage proteins from core eudicots to gymnosperms were included in the analysis. The two AP3-like proteins from *Fragaria vesca* (FvH4_1g12260 and FvH4_2g38970) are represented in bold types. Four *PISTILLATA* genes were used as outgroup. Numbers next to the nodes are bootstrap values from 1000 pseudo-replicates. The protein sequences were obtained from GenBank (see Accession numbers section).

Supplementary Figure S2

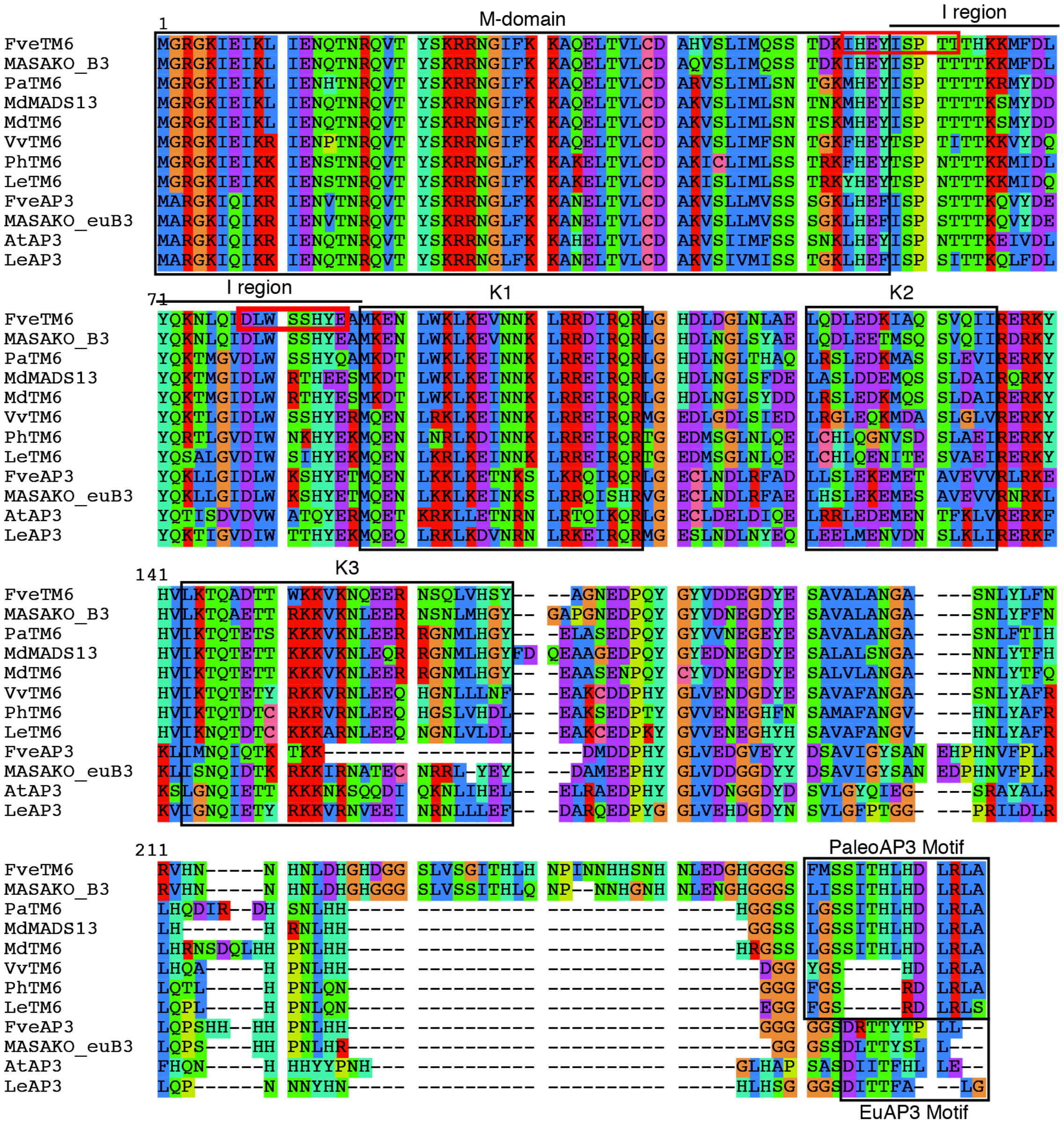

Supplementary Figure S2. Alignment of AP3- and TM6-like proteins. Eight TM6- and four AP3-like proteins were selected for the alignment. The M- and K-domain characteristics of MIKC-type MADS transcription factors are boxed. PaleoAP3 and EuAP3 motives are located at the carboxyl end of the TM6- and AP3-like proteins. Red squares mark the region where the sgRNAs were designed for *F. vesca TM6* (*FveTM6*). sgRNA1 is located spanning the M-domain and the I region. sgRNA2 is located at the I region. FveTM6 and FveAP3 (*F. vesca*), MASAKO B3 and MASAKO euB3 (*Rosa rugosa*), PaTM6 (*Prunus avium*), MdMADS13 and MdTM6 (*Malus* × *domestica*), VvTM6 (*Vitis vinifera*), PhTM6 (*Petunia* × *hybrid*), LeTM6 and LeAP3 (*Solanum lycopersicum*), AtAP3 (*Arabidopsis thaliana*). The protein sequences were obtained from GenBank (see Accession numbers section).

Supplementary Figure S3

```
                                   61
F. vesca cv. Hawaii 4              TATTCGAAGCGACGAAATGGGATCTTCAAGAAGGCTCAAGAGCTCACGGTTCTGTGTGAT
F. vesca cv. RV                    TATTCGAAGCGACGAAATGGGATCTTCAAGAAGGCTCAAGAGCTCACGGTTCTGTGTGAT
F. × ananassa cv. Camarosa #1      TATTCGAAGCGACGAAATGGGATCTTCAAGAAGGCTCAAGAGCTCACGGTTCTGTGTGAT
F. × ananassa cv. Camarosa #2      TATTCGAAGCGACGAAATGGGATCTTCAAGAAGGCTCAAGAGCTCACGGTTCTGTGTGAT
F. × ananassa cv. Camarosa #3      TATTCGAAGCGACGAAATGGGATCATCAAGAAGGCTCAGGAGCTCACGGTTCTTTGTGAT
F. × ananassa cv. Camarosa #4      TATTCGAAGCGACGAAATGGGATCTTCAAGAAGGCTCAAGAGCTCACGGTTATGTGTGAT
F. × ananassa cv. Camarosa #5      TATTCGAAGCGACGAAATGGGATCTTCAAGAAGGCTCAAGAGCTCACGGTTCTGTGTGAT
                                   ************************ ************* ************ * ******

                                   121                                      PAM     sgRNA1
F. vesca cv. Hawaii 4              GCTCATGTCTCCCTCATCATGCAGTCCTCCACTGATAAAATCCACGAGTATATTAGCCCT
F. vesca cv. RV                    GCTCATGTCTCCCTCATCATGCAGTCCTCCACTGATAAAATCCACGAGTATATTAGCCCT
F. × ananassa cv. Camarosa #1      GCTCATGTCTCCCTCATCATGCAGTCCTCCACTGATAAAATCCACGAGTATATTAGCCCT
F. × ananassa cv. Camarosa #2      GCTCATGTCTCCCTCATCATGCAGTCCTCCACTGATAAAATCCACGAGTATATTAGCCCT
F. × ananassa cv. Camarosa #3      GCTCAGGTCTCCCTCATCATGCAGTCCTCCACTAATAGAATCCACGAGTATATTAGCCCT
F. × ananassa cv. Camarosa #4      GCTCAGGTTTCCCTCATCATGCAGTCCTCCACTGATAAAATCCACGAGTATATTAGCCCT
F. × ananassa cv. Camarosa #5      GCTCAGGTCTCCCTCATCATGCAGTCCTCCACTGATAAAATCCACGATTATATTAGCCCT
                                   ***** ** ************************ *** ********* ************

                                   181
F. vesca cv. Hawaii 4              ACCACTACGTACGTCCAACATCTCTAGCTAGCTACTCTTTATTTATGATCTTTTGTTCAC
F. vesca cv. RV                    ACCACTACGTACGTCCAACATCTCTAGCTAGCTACTCTTTATTTATGATCTTTTGTTCAC
F. × ananassa cv. Camarosa #1      ACCACTACGTACGTCCAACATCTCTAGCTAGCTACTCTTTATTTATGATCTTTTGTTCAC
F. × ananassa cv. Camarosa #2      ACCACTACGTACGTCCAACATCTCTAGCTAGCTACTCTTTATTTATGATCTTTTGTTCAC
F. × ananassa cv. Camarosa #3      ACCACTACGTACGTCCAACATCTCTAGCTAGCTACTCTTTATTTATGATCTTTTGTTCAC
F. × ananassa cv. Camarosa #4      ACCACTACGTACGTCCAACATCTCTAGCTAGCTACTCTTTATTTATGATCTTTTGTTCAC
F. × ananassa cv. Camarosa #5      ACCACTACGTACGTCCAACATCTCTAGCTAGCTACTCTTTATTTATGATCTTTTGTTCAC
                                   ************************************************************

                                   241
F. vesca cv. Hawaii 4              TTTTGATCTTTGCTTGATAATTCCATATATAGAACTGATACGGATGTAAATCAGGCACAA
F. vesca cv. RV                    TTTTGATCTTTGCTTGATAATTCCATATATAGAACTGATACGGATGTAAATCAGGCACAA
F. × ananassa cv. Camarosa #1      TTTTGATCTTTGCTTGATAATTCCATATATAGAACTGATACGGATGTAAATCAGGCACAA
F. × ananassa cv. Camarosa #2      TTTTGATCTTTGCTTGATAATTCCATATATAGAACTGATACGGATGTAAATCAGGCACAA
F. × ananassa cv. Camarosa #3      TTTTGATCTTTGTTTGATTCCATATATAGAACTG---ATACGGATGTAAATCAGGCACAA
F. × ananassa cv. Camarosa #4      TTTTGATCTTTGGTTGATTCCATATATAGAACTGATGATACGGATGTAAATCAGGCACAA
F. × ananassa cv. Camarosa #5      TTTTGATCTTTGCTTGATTCCATATATAGAACTG---ATACGGATGTAAATCAGGCACAA
                                   ************ *****            *      ***********************

                                   301                                            sgRNA2      PAM
F. vesca cv. Hawaii 4              GAAGATGTTTGATCTCTACCAAAAGAATTTGCAGATCGATCTATGGAGCTCGCACTACGA
F. vesca cv. RV                    GAAGATGTTTGATCTCTACCAAAAGAATTTGCAGATCGATCTATGGAGCTCGCACTACGA
F. × ananassa cv. Camarosa #1      GAAGATGTTTGATCTCTACCAAAAGAATTTGCAGATCGATCTATGGAGCTCGCACTACGA
F. × ananassa cv. Camarosa #2      GAAGATGTTTGATCTCTACCAAAAGAATTTGCAGATCGATCTATGGAGCTCGCACTACGA
F. × ananassa cv. Camarosa #3      GAAGGTGTTTGATCTCTACCAGAAGAATTTGCAGATCGATCTATGGAGCTCGCACTACGA
F. × ananassa cv. Camarosa #4      GAAGATGTATGATCTCTACCAGAAGAATTTGCAGATCGATCTATGGAGCTCGCACTACGA
F. × ananassa cv. Camarosa #5      GAAGATGTTTGATCTCTACCAGAAGAATTTGCAGATCGATCTATGGAGCTCGCACTACGA
                                   **** *** ************ **************************************

                                   361
F. vesca cv. Hawaii 4              GGTGATTAAAGTTAACCAAAGGAGTCTGTCAATTTTTT----------TTATTCTATAAA
F. vesca cv. RV                    GGTGATTAAAGTTAACCAAAGGAGTCTGTCAATTTTTT----------TTATTCTATAAA
F. × ananassa cv. Camarosa #1      GGTGATTAAAGTTAACCAAAGGAGTCTGTCAATTTTTT----------TTATTCTATAAA
F. × ananassa cv. Camarosa #2      GGTGATTAAAGTTAACCAATGGAGTCTGTCAATTTTTT----------TTATTCTATAAA
F. × ananassa cv. Camarosa #3      GGTGATTAAAGTTAACCAAAGGAGTCTGTCAATTTTATTTATTTATTTTTATTCTATATA
F. × ananassa cv. Camarosa #4      GGTGATTAAAGTTAACCAAAGGATATAGAAAATAGTTGTA--------------------
F. × ananassa cv. Camarosa #5      GGTGATTAAAGTTAACCAAAGGATATAGAAAATATTTGTA--------------------
                                   ******************* ***    *  ***  *

                                   421
F. vesca cv. Hawaii 4              CAAAATATTTGTAGAGTTTCTGTATGTGTTAATTTTGTTTGCTGATTTTGGTGGA
F. vesca cv. RV                    CAAAATATTTGTAGAGTTTCTGTATGTGTTAATTTTGTTTGCTGATTTTGGTGGA
F. × ananassa cv. Camarosa #1      CAAAATATTTGTAGAGTTTCTGTATGTGTTAATTTTGTTTGCTGATTTTGGTGGA
F. × ananassa cv. Camarosa #2      CAAAATATTTGTAGAGTTTCTGTATGTGTTAATTTTGTTTGCTGATTTTGGTGGA
F. × ananassa cv. Camarosa #3      GAAAATATTTGGAGAGTTTTTGTATGTGTTAATTTTGTTTGCTGATTTTGGTGGA
F. × ananassa cv. Camarosa #4      -------------GAGTTTCTGTATGTGTTAATTTTGTTTGCTGATTTTGGTGGA
F. × ananassa cv. Camarosa #5      -------------GAGTTTCTGTATGTGTTAATTTTGTTTGCTGATTTTGGTGGA
                                                ****** ***********************************
```

Supplementary Figure S3. Alignment of *TM6* sequences from *F. vesca* and *F.* × *ananassa*. PCR flanking the two target sites (primers P180 and P181; Supplemental Table 3) for *TM6* was performed, purified, cloned and sequenced by the Sanger method for *F. vesca* cv. Hawaii 4, *F. vesca* cv. Reine des Vallées (RV), and *F.* × *ananassa* cv. Camarosa. The aligned region spans from the position 61 after the start codon, to the nucleotide 475, based on the *TM6* sequence in *F. vesca.* Exons are delimited with a black line; red font: sgRNAs; grey background: PAM; green background: synonymous

polymorphisms; red background: non-synonymous polymorphisms; asterisks: conserved nucleotides.

Supplementary Figure S4

```
#Alleles
          33
Control
  #1    QELTVLCDAHVSLIMQSSTDKIHE-YISPTTTHKKMFDLYQKNLQIDLWSSHYEAMKENLWKLKEVNN
  #2    QELTVLCDAHVSLIMQSSTDKIHE-YISPTTTHKKMFDLYQKNLQIDLWSSHYEAMKENLWKLKEVNN
  #3    QELTVLCDAQVSLIMQSSTNRIHE-YISPTTTHKKVFDLYQKNLQIDLWSSHYEAMKENLWKLKEVNN
  #4    QELTVMCDAQVSLIMQSSTDKIHE-YISPTTTHKKMYDLYQKNLQIDLWSSHYEAMKENLWKLKEVNN
  #5    QELTVLCDAQVSLIMQSSTDKIHD-YISPTTTHKKMFDLYQKNLQIDLWSSHYEAMKENLWKLKEVNN

tm6-1
  #1    QELTVLCDAHVSLIMQSSTDKIHE-----------------------------AMKENLWKLKEVNN -29
  #2    QELTVLCDAHVSLIMQSSTDKIHETRQ*                                        PTC
  #3-A  QELTVLCDAQVSLIMQSSTNRIHD*                                           PTC
  #3-B  QELTVLCDAQVSLIMQSSTNRIHE---SPTTTHKKVFDLYQKNLQIDLWSRGNERELVETEGG*    PTC
  #4-A  QELTVMCDAQVSLIMQSSTDKIHE-Y*                                         PTC
  #4-B  QELTVMCDAQVSLIMQSSTDKIHEVY*                                         PTC
  #5-A  QELTVLCDAQVSLIMQSSTDKIHD-YISPTTTHKKMFDLYQKNLQIDLWSSHTRQ*            PTC
  #5-B  QELTVLCDAQVSLIMQSSTDKIHD-YISPTTTHKKMFDLYQKNLQIDLWSSPRGNERELVETEGG*  PTC
  #5-C  QELTVLCDAQVSLIMQSSTDKIHD-YISPTTTHKKMFDLYQKNLQIDLWSSHYEAMKENLWKLKEVNN wt

tm6-7
  #1    QELTVLCDAHVSLIMQSSTDKIHE-Y---------------------------EAMKENLWKLKEVNN -27
  #2    QELTVLCDAHVSLIMQSSTDKIHETRQ*                                        PTC
  #3    QELTVLCDAQVSLIMQSSTNRIHE-Y---------------------------EAMKENLWKLKEVNN -27
  #4-A  QELTVMCDAQVSLIMQSSTDKIHE-Y---------------------------EAMKENLWKLKEVNN -27
  #4-B  QELTVMCDAQVSLIMQSSTDKIHE-YISPTTTHKKMYDLYQKNLQIDLWSSH-EAMKENLWKLKEVNN -1
  #5-A  QELTVLCDAQVSLIMQSSTDKIHD-YISPTTTHKKMFDLYQKNLQIDLWSSHTRQ*            PTC
  #5-B  QELTVLCDAQVSLIMQSSTDKIHD-YISPTTTHKKMFDLYQKNLQIDLWSSHYEAMKENLWKLKEVNN wt

tm6-9
  #1    QELTVLCDAHVSLIMQSSTDKIHEVY*                                         PTC
  #2    QELTVLCDAHVSLIMQSSTDKIHETRQ*                                        PTC
  #3-A  QELTVLCDAQVSLIMQSSTNRIH--------VRTRRCLISTRRICRSIYGARYEAMKENLWKLKEVNN 21 aas subst/-7
  #3-B  QELTVLCDAQVSLIMQSSTNRILAL----PLRTRRCLISTRRICRSIYGARTRQ*             PTC
  #4-A  QELTVMCDAQVSLIMQSSTDSTRRCMIST------------------------EAMKENLWKLKEVNN 9 aas subst/-23
  #4-B  QELTVMCDAQVSLIMQSSTDKIHD--ISPTTTHKKMYDLYQKNLQIDLWSSH-EAMKENLWKLKEVNN 1 aa subst/-2
  #4-C  QELTVMCDAQVSLIMQSSTDKIHE-Y---------------------------EAMKENLWKLKEVNN -27
  #5-A  QELTVLCDAQVSLIMQSSTDKIHD-YISPTTTHKKMFDLYQKNLQIDLWSSHLRGNERELVETEGG* PTC
  #5-B  QELTVLCDAQVSLIMQSSTDKIHD-YISPTTTHKKMFDLYQKNLQIETYS*                 PTC
  #5-C  QELTVLCDAQVSLIMQSSTDKIHD-YISPTTTHKKMFDLYQKNLQIDLWSSHYEAMKENLWKLKEVNN wt
```

Supplementary Figure S4. Alignment of TM6 predicted amino acid sequences. TM6 protein sequence from amino acid 33 to 99 in control is aligned with the protein sequences of the *tm6* mutant lines. Red and bold fonts indicate CRISPR/Cas9-induced variants. Red asterisk: premature termination codon (PTC). Information about the amino acid modification is included after the protein sequence.

Supplementary Figure S5

A

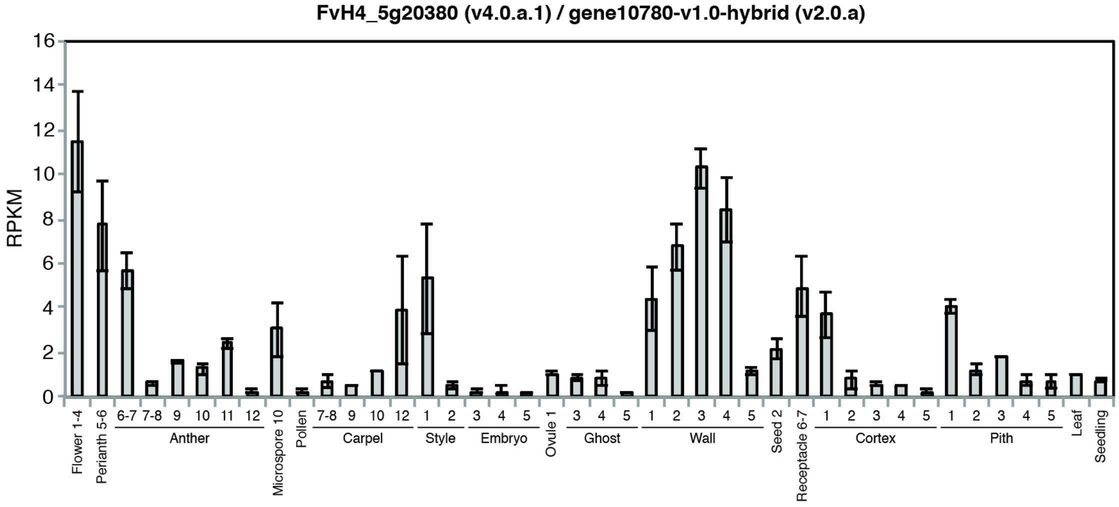

B

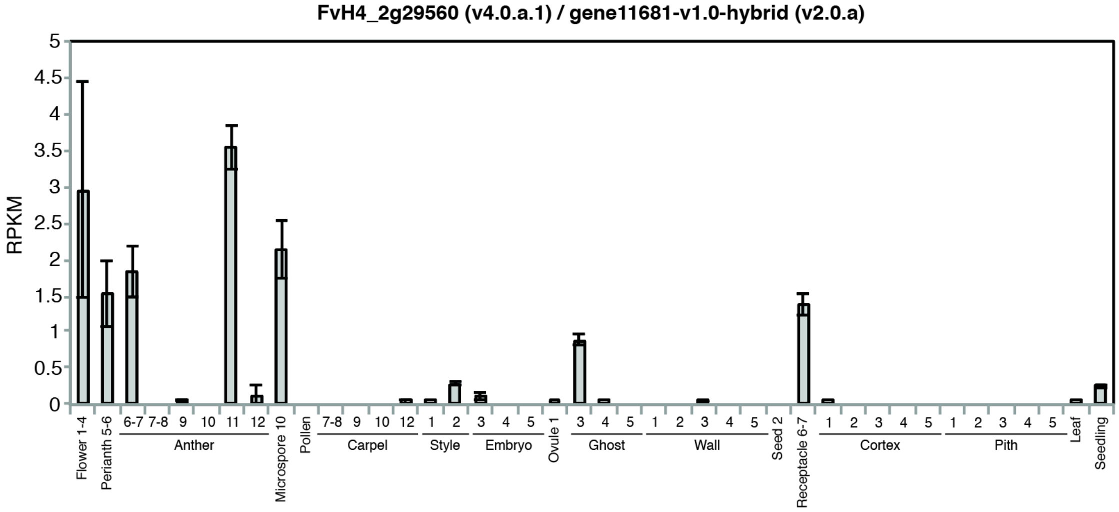

Supplementary Figure S5. Expression analysis of two putative off-targets. Expression of FvH4_5g20380 and FvH4_2g29560 was analyzed using the eFP browser for *F. vesca* (Hawkins *et al.*, 2017). Expression data from the flower and fruit stages were obtained from Hollender *et al*. (2014) and Kang *et al*. (2013) respectively. All stage numbering follows Hollender *et al*. (2011).

Supplementary Figure S6

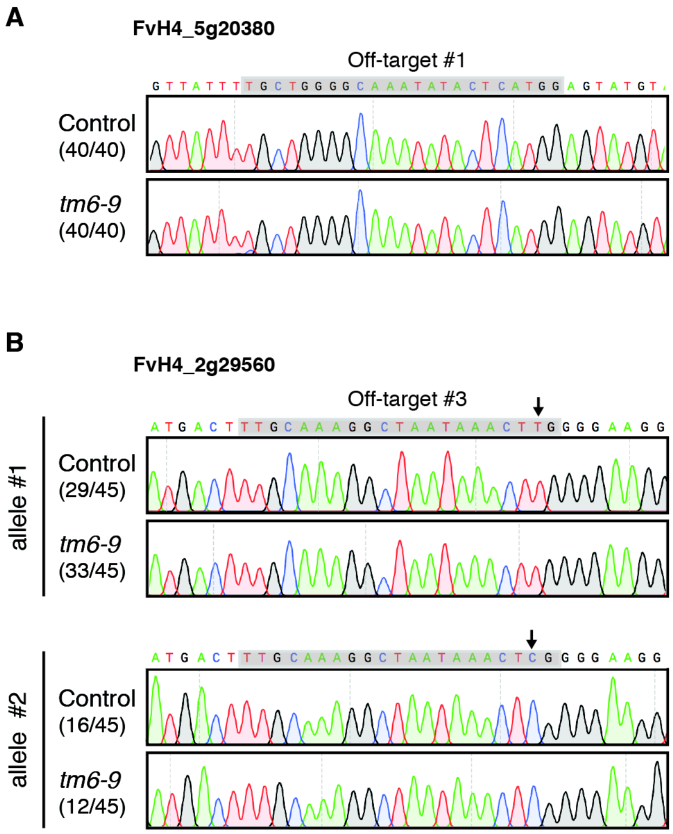

Supplementary Figure S6. Sequence analyses of two putative off-targets. Spanning region of the putative off-targets #1 (2nd exon of FvH4_5g20380) (A), and #3 (4th exon of FvH4_2g29560) (B) for the sgRNA1 were amplified and Sanger sequenced. No variation in the sequence was found between the control and the *tm6-9* mutant line. Off-target sequences are boxed in grey; frequency of each sequence is indicated in parenthesis; arrow indicates a single nucleotide polymorphism detected at the off-target #3, indicating that FvH4_2g29560 contains at least two alleles in *F.* × *ananassa* cv. Camarosa.

Supplementary Figure S7

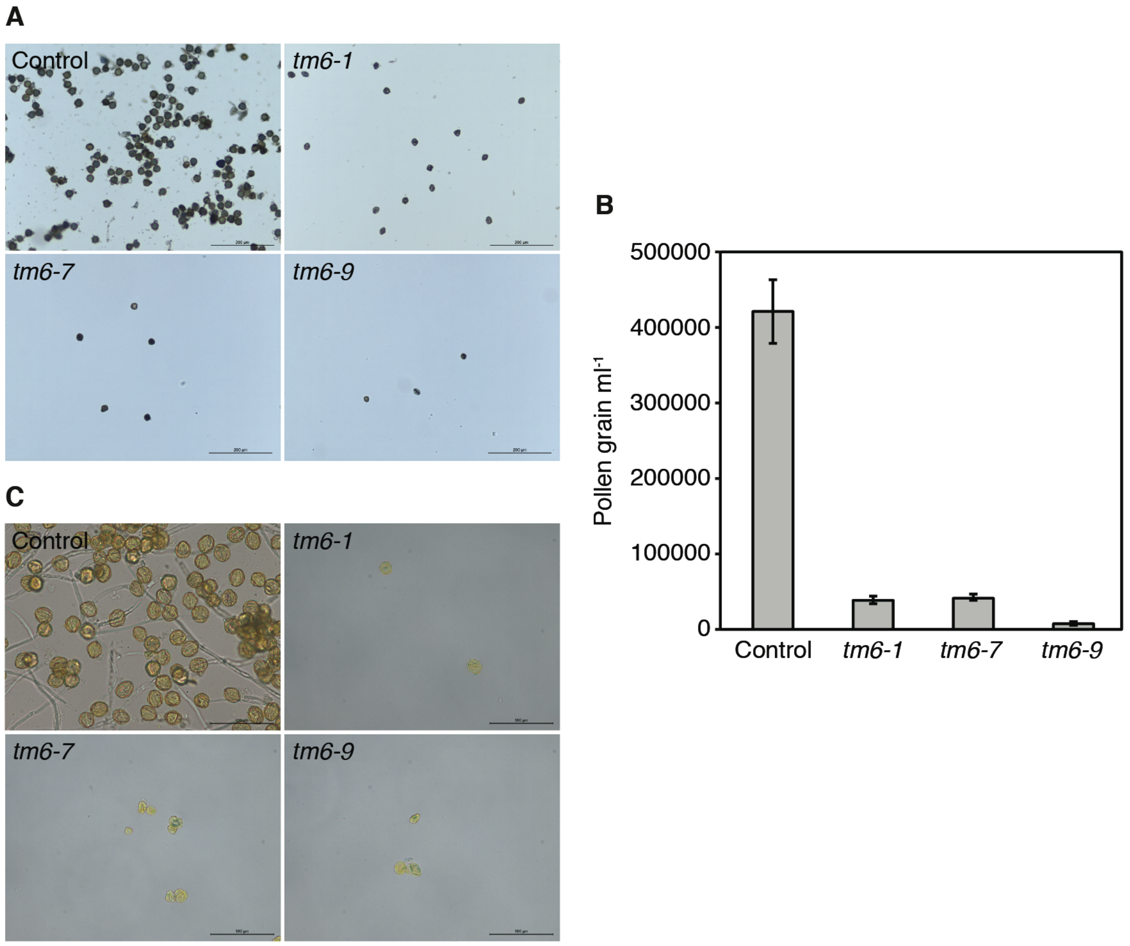

Supplementary Figure S7. Pollen yield quantification and germination assay. (A) Pictures of pollen grains stained with acetocarmine. (B) Quantification of pollen amount using the Neubauer chamber. Error bars denote the standard deviation (s.d.) of three biological replicates. (C) Pollen germination assay.

Supplementary Figure S8

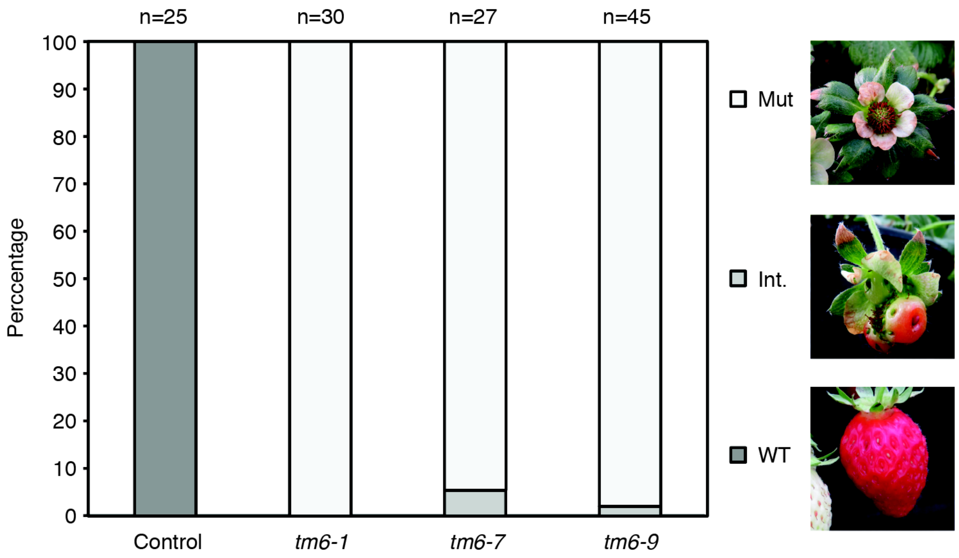

Supplementary Figure S8. Fruit phenotype quantification. Chart showing the percentage of fruits with mutant, intermediate (Int.) and *wild-type* phenotype in control and *tm6* lines. Fruits with partial receptacle enlargement were considered to have an intermediate phenotype. Numbers of fruits analyzed for each genotype are indicated above the bars.

Supplementary Table S1

	Sequence	CFD score	Position (Strawberry Genome v4.0.a1 chromosomes/maker standard CDS)
sgRNA1	TGGTAGGGCTAATATACTCG *TGG*		Fvb1_v4.0.a1: 6715277 - 6715299 (+) / FvH4_1g12260 (1st exon)
Off-target #1	TG**C**T**G**GGGC**A**AATATACTC**A** *TGG*	0.222527	Fvb5_v4.0.a1: 12055720 - 12055742 (+) / FvH4_5g20380 (2nd exon)
Off-target #2	TGGT**T**GGGCTAATATA**TA**C**A** *TGG*	0.106061	Fvb2_v4.0.a1: 25934976 - 25934954 (-) / intergenic region (putative promoter of FvH4_2g35080 (279 nt upstream of start codon) and/or FvH4_2g35070 (2593 nt upstream of start codon)
Off-target #3	TG**CA**A**A**GGCTAATA**A**ACTCG ***G**GG*	0.106034	Fvb2_v4.0.a1: 22903461 - 22903439 (-) / FvH4_2g29560 (4th exon)
Off-target #4	TG**T**T**G**GGGCTAAT**C**TA**T**TCG ***C**GG*	0.036000	Fvb4_v4.0.a1: 19433168 - 19433146 (-) / intergenic region
Off-target #5	TGG**G**AG**T**GCTAATATA**G**T**G**G *TGG*	0.002626	Fvb4_v4.0.a1: 10677003 - 10677025 (+) / intergenic region
sgRNA2	TCTATGGAGCTCGCACTACG *AGG*		Fvb1_v4.0.a1: 6715121 - 6715099 (-) / FvH4_1g12260 (2nd exon)
Off-target #1	TC**G**A**C**GGAGCT**A**GCACTAC**T** ***C**GG*	0.250000	Fvb3_v4.0.a1: 18665022 - 18665044 (+) / intergenic region
Off-target #2	T**A**TATGGA**C**CTCG**TT**CTACG ***G**GG*	0.057436	Fvb5_v4.0.a1: 27110536 - 27110514 (-) / intergenic region

Supplementary Table S1. Off-target analysis for sgRNA1 and sgRNA2. Sequences, Cutting Frequency Determination (CFD) score (Doench *et al.*, 2016), and position in the *F. vesca* v4.0.a1 reference genome (Edger *et al.*, 2018) are displayed. CFD score is predictive of off-target potential of sgRNA:DNA interactions. Off-targets are ranked by CFD score from most to least likely. Mismatches compared with the sgRNA sequence are shown in bold type. Off-targets located within coding sequences (CDS) are marked in grey.

Supplementary Table S2.

Oligo	Sequence (5' -> 3')	Amplicon size	Purpose
P131	GATTGGGTAGGGCTAATATACTCG	24 bp	sgRNA1
P132	AAACCGAGTATATTAGCCCTACCC		
P133	GATTGCTATGGAGCTCGCACTACG	24 bp	sgRNA2
P134	AAACCGTAGTGCGAGCTCCATAGC		
P177	ATTGCCCGGGAGCTTCGTTGAAC	600 bp	Cloning sgRNAs into the final vector
P179	GCCGTCTAGATGATGGATATCTGC		
P443	ATCACCACAGCAACCACAAC	85 bp	qRT-PCR *FaTM6*
P444	AGGCGGAGATCATGGAGATG		
P476	TCAGATTCAAACCAAGACGAAA	97 bp	qrt-PCR *FaAP3*
P477	AGCCAATGACTGCAGAATCA		
FaCHP1-F	TGCATATATCAAGCAACTTTACACTGA	91 bp	qRT-PCR *CHP1* (housekeeping)
FaCHP1-R	ATAGCTGAGATGGATCTTCCTGTGA		
P180	AGCTGATTGAGAACCAGACGA	512 bp	*FveTM6* and *FaTM6* alleles characterization. sgRNA1-2/Cas9-mediated mutagenesis detection
P181	CGAGTCATCAAATGGCCAAAC		
P248	GAGATCGTGTGGGATAAGGG	717 bp	Cas9 detection
P297	GATATTCTCGGCCTGCTCTC		
P445	TCGTCGGCAGCGTCAGATGTGTATAAGAGACAGTATTCGAAGCGACGAAATGG	472 bp	*FveTM6* amplicon sequencing (NGS) gDNA
P446	GTCTCGTGGGCTCGGAGATGTGTATAAGAGACAGTCCACCAAAATCAGCAAACA		
P445	TCGTCGGCAGCGTCAGATGTGTATAAGAGACAGTATTCGAAGCGACGAAATGG	314 bp	*FveTM6* amplicon sequencing (NGS) gDNA
P475	GTCTCGTGGGCTCGGAGATGTGTATAAGAGACAGTCCTCAGCTTATTGTTAACCTC		
P546	TCTGTAGCCCACAAAACAGC	462bp	Off-target #1 located at the 2nd exon of FvH4_5g20380

P547	CCTATGGCAAAGGCAGAAAG		
P544	TAGGCGAGAAGGGATTGTCA	389bp	Off-target #3 located at the 4th exon of FvH4_2g29560
P545	TAGCTTCCCCTGCATTGTTC		

Supplementary Table S2. List of oligonucleotides used in this study.

References

Doench JG, Fusi N, Sullender M, *et al.* 2016. Optimized sgRNA design to maximize activity and minimize off-target effects of CRISPR-Cas9. Nature biotechnology **34**, 184–191.

Edger PP, Vanburen R, Colle M, *et al.* 2018. Single-molecule sequencing and optical mapping yields an improved genome of woodland strawberry (Fragaria vesca) with chromosome-scale contiguity. GigaScience **7**, 1–7.

Hawkins C, Caruana J, Li J, Zawora C, Darwish O, Wu J, Alkharouf N, Liu Z. 2017. An eFP browser for visualizing strawberry fruit and. Horticulture research, 1–8.

Hollender CA, Geretz AC, Slovin JP, Liu Z. 2011. Flower and early fruit development in a diploid strawberry, Fragaria vesca. Planta **235**, 1123–1139.

Hollender CA, Kang C, Darwish O, Geretz A, Matthews BF, Slovin J, Alkharouf N, Liu Z. 2014. Floral transcriptomes in woodland strawberry uncover developing receptacle and anther gene networks. Plant physiology.

Kang C, Darwish O, Geretz A, Shahan R. 2013. Genome-Scale Transcriptomic Insights into Early-Stage Fruit Development in Woodland Strawberry Fragaria vesca. The Plant cell.
