# Supplementary material for: Functional analysis of the TM6 MADS-box gene in the octoploid strawberry by CRISPR/Cas9-directed mutagenesis
Source: J Exp Bot. 2018 Nov 13;70(3):885–95. doi: 10.1093/jxb/ery400 (PMC6363087; doi:10.1093/jxb/ery400)
Supplement: Supplementary Figure Legends [file ery400_suppl_supplementary_figure_legends.docx]

**Supplementary Figure S1.** Neighbor-Joining Analysis of TM6 and euAP3 lineage proteins. Representative AP3 lineage proteins from core eudicots to gymnosperms were included in the analysis. The two AP3-like proteins from *Fragaria vesca* (FvH4_1g12260 and FvH4_2g38970) are represented in bold types. Four *PISTILLATA* genes were used as outgroup. Numbers next to the nodes are bootstrap values from 1000 pseudo-replicates. The protein sequences were obtained from GenBank (see Accession numbers section).

**Supplementary Figure S2.** Alignment of AP3- and TM6-like proteins. Eight TM6- and four AP3-like proteins were selected for the alignment. The M- and K-domain characteristics of MIKC-type MADS transcription factors are boxed. PaleoAP3 and EuAP3 motives are located at the carboxyl end of the TM6- and AP3-like proteins. Red squares mark the region where the sgRNAs were designed for *F. vesca TM6* (*FveTM6*). sgRNA1 is located spanning the M-domain and the I region. sgRNA2 is located at the I region. FveTM6 and FveAP3 (*F. vesca*), MASAKO B3 and MASAKO euB3 (*Rosa rugosa*), PaTM6 (*Prunus avium*), MdMADS13 and MdTM6 (*Malus* × *domestica*), VvTM6 (*Vitis vinifera*), PhTM6 (*Petunia* × *hybrid*), LeTM6 and LeAP3 (*Solanum lycopersicum*), AtAP3 (*Arabidopsis thaliana*). The protein sequences were obtained from GenBank (see Accession numbers section).

**Supplementary Figure S3.** Alignment of *TM6* sequences from *F. vesca* and *F.* × *ananassa*. PCR flanking the two target sites (primers P180 and P181; Supplemental Table 3) for *TM6* was performed, purified, cloned and sequenced by the Sanger method for *F. vesca* cv. Hawaii 4, *F. vesca* cv. Reine des Vallées (RV), and *F.* × *ananassa* cv. Camarosa. The aligned region spans from the position 61 after the start codon, to the nucleotide 475, based on the *TM6* sequence in *F. vesca.* Exons are delimited with a black line; red font: sgRNAs; grey background: PAM; green background: synonymous polymorphisms; red background: non-synonymous polymorphisms; asterisks: conserved nucleotides.

**Supplementary Figure S4.** Alignment of TM6 predicted amino acid sequences. TM6 protein sequence from amino acid 33 to 99 in control is aligned with the protein sequences of the *tm6* mutant lines. Red and bold fonts indicate CRISPR/Cas9-induced variants. Red asterisk: premature termination codon (PTC). Information about the amino acid modification is included after the protein sequence.

**Supplementary Figure S5.** Expression analysis of two putative off-targets. Expression of FvH4_5g20380 and FvH4_2g29560 was analyzed using the eFP browser for *F. vesca* (Hawkins *et al.*, 2017). Expression data from the flower and fruit stages were obtained from Hollender *et al*. (2014) and Kang *et al*. (2013) respectively. All stage numbering follows Hollender *et al*. (2011).

**Supplementary Figure S6.** Sequence analyses of two putative off-targets. Spanning region of the putative off-targets #1 (2nd exon of FvH4_5g20380) (A), and #3 (4th exon of FvH4_2g29560) (B) for the sgRNA1 were amplified and Sanger sequenced. No variation in the sequence was found between the control and the *tm6-9* mutant line. Off-target sequences are boxed in grey; frequency of each sequence is indicated in parenthesis; arrow indicates a single nucleotide polymorphism detected at the off-target #3, indicating that FvH4_2g29560 contains at least two alleles in *F. × ananassa* cv. Camarosa.

**Supplementary Figure S7.** Pollen yield quantification and germination assay. (A) Pictures of pollen grains stained with acetocarmine. (B) Quantification of pollen amount using the Neubauer chamber. Error bars denote the standard deviation (s.d.) of three biological replicates. (C) Pollen germination assay.

**Supplementary Figure S8.** Fruit phenotype quantification. Chart showing the percentage of fruits with mutant, intermediate (Int.) and *wild-type* phenotype in control and *tm6* lines. Fruits with partial receptacle enlargement were considered to have an intermediate phenotype. Numbers of fruits analyzed for each genotype are indicated above the bars.

**Supplementary Table S1.** Off-target analysis for sgRNA1 and sgRNA2. Sequences, Cutting Frequency Determination (CFD) score (Doench *et al.*, 2016), and position in the *F. vesca* v4.0.a1 reference genome (Edger *et al.*, 2018) are displayed. CFD score is predictive of off-target potential of sgRNA:DNA interactions. Off-targets are ranked by CFD score from most to least likely. Mismatches compared with the sgRNA sequence are shown in bold type. Off-targets located within coding sequences (CDS) are marked in grey.

**Supplementary Table S2.** List of oligonucleotides used in this study.

**Supplementary Table S3.** Analysis of high-throughput sequencing of amplicons of *TM6* cDNA and genomic DNA. *TM6* sequences flanking the two target sites were obtained from cDNA from petals and stamens of *F.* × *ananassa* cv. Camarosa (sheets 1 and 2), and from gDNA from leaves of control and *tm6* lines (sheets 3-6). % Prevalence indicates the presence of each cluster obtained by *de-novo* assembly.
